# The Reconfigurable Maze Provides Flexible, Scalable, Reproducible, and Repeatable Tests

**DOI:** 10.1016/j.isci.2019.100787

**Published:** 2019-12-19

**Authors:** Satoshi Hoshino, Riku Takahashi, Kana Mieno, Yuta Tamatsu, Hirotsugu Azechi, Kaoru Ide, Susumu Takahashi

**Affiliations:** 1 Laboratory of Cognitive and Behavioral Neuroscience, Graduate School of Brain Science, Doshisha University, Kyotanabe City, Kyoto 610-0394, Japan

**Keywords:** Neuroscience, Behavioral Neuroscience, Techniques in Neuroscience

## Abstract

Multiple mazes are routinely used to test the performance of animals because each has disadvantages inherent to its shape. However, the maze shape cannot be flexibly and rapidly reproduced in a repeatable and scalable way in a single environment. Here, to overcome the lack of flexibility, scalability, reproducibility, and repeatability, we develop a reconfigurable maze system that consists of interlocking runways and an array of accompanying parts. It allows experimenters to rapidly and flexibly configure a variety of maze structures along the grid pattern in a repeatable and scalable manner. Spatial navigational behavior and hippocampal place coding were not impaired by the interlocking mechanism. As a proof-of-principle demonstration, we demonstrate that the maze morphing induces location remapping of the spatial receptive field. The reconfigurable maze thus provides flexibility, scalability, repeatability, and reproducibility, therefore facilitating consistent investigation into the neuronal substrates for learning and memory and allowing screening for behavioral phenotypes.

## Highlights

• The reconfigurable maze enables flexibly in building a variety of maze paths
• The maze ensures reproducibility, repeatability, and scalability
• The maze does not impair spatial navigational behavior or neuronal activity
• Various tests of learning and memory can be conducted in a single environment

## Graphical Abstract

**Figure:**
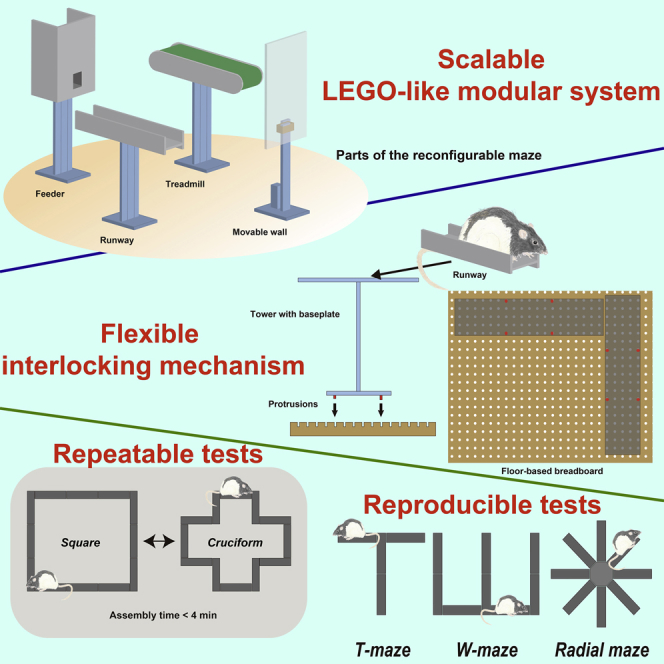

## Introduction

Several shapes of mazes such as the T-maze (Small, 1901, Dudchenko, 2001), plus maze (Olton and Feustle, 1981), radial arm maze (Olton and Samuelson, 1976, Olton et al., 1978), and figure-8 maze (Wood et al., 2000) have been designed as behavioral tests to assess the performance of working (Dudchenko, 2004), reference (Olton and Paras, 1979, Xu et al., 2019) and episodic-like memory (Babb and Crystal, 2005), and spatial navigation (O'Keefe and Nadel, 1978), as well as for studying anxiety (Walf and Frye, 2007). As individual tests of learning and memory, all have pros and cons (Andersen et al., 2009). To compensate for the disadvantages, evidence from a battery of maze tests is usually accumulated to understand specific learning and memory (Tecott and Nestler, 2004). However, each test is often conducted in a different real or virtual experimental room because conventional mazes cannot be easily rebuilt in a systematically arranged manner. The details of the maze structure, including shape, coordination, and dimensions, are crucial aspects of the test results, whereas the structures themselves cannot be precisely reproduced in different laboratories in a repeatable way. Thus, conventional maze tests lack reproducibility and repeatability.

Place cells found in the hippocampus encode an animal's location and are deeply involved in spatial navigation ability (O'Keefe and Nadel, 1978). Place coding responds dramatically to changes in the surrounding environment (Muller and Kubie, 1987), suggesting that even if an animal experiences a single maze set inside different rooms, the hippocampus may generate different cognitive maps for each room, despite the mazes having identical shapes. On the other hand, conventional visual-based virtual reality (VR) systems can realize unlimited shapes of mazes in visual space. However, the lack of non-visual sensory feedback in passive VR results in altered neuronal responses. Furthermore, it forces animals to be partially or fully immobilized because one of the best applications of VR is to monitor neuronal activity from two-photon calcium imaging during the spatial navigation of head-fixed animals. For instance, a fraction of place cells reduced their firings in the spatial receptive field apparently because of the loss of vestibular feedback in the VR (Aronov and Tank, 2014). These altered neuronal responses impede detailed interpretations of the learning and memory mechanisms involved in maze tests.

Here we develop a maze system that overcomes most of these limitations by allowing the reconfiguration of the shape of the maze in a single physical environment. We demonstrate the use of the reconfigurable maze system to replicate existing mazes, including the T-maze, plus maze, W-maze, figure-8 maze, and radial arm maze. We also investigate the spatial navigational behavior and coding of the hippocampal place cells of rats to address concerns over possible distortions caused by the interlocking mechanisms and perform a novel experiment that morphs the shape of the maze. Together, these contribute to deciphering the neuronal underpinning of spatial navigation.

## Results

### Implementation of the Reconfigurable Maze

**Figure 1:**
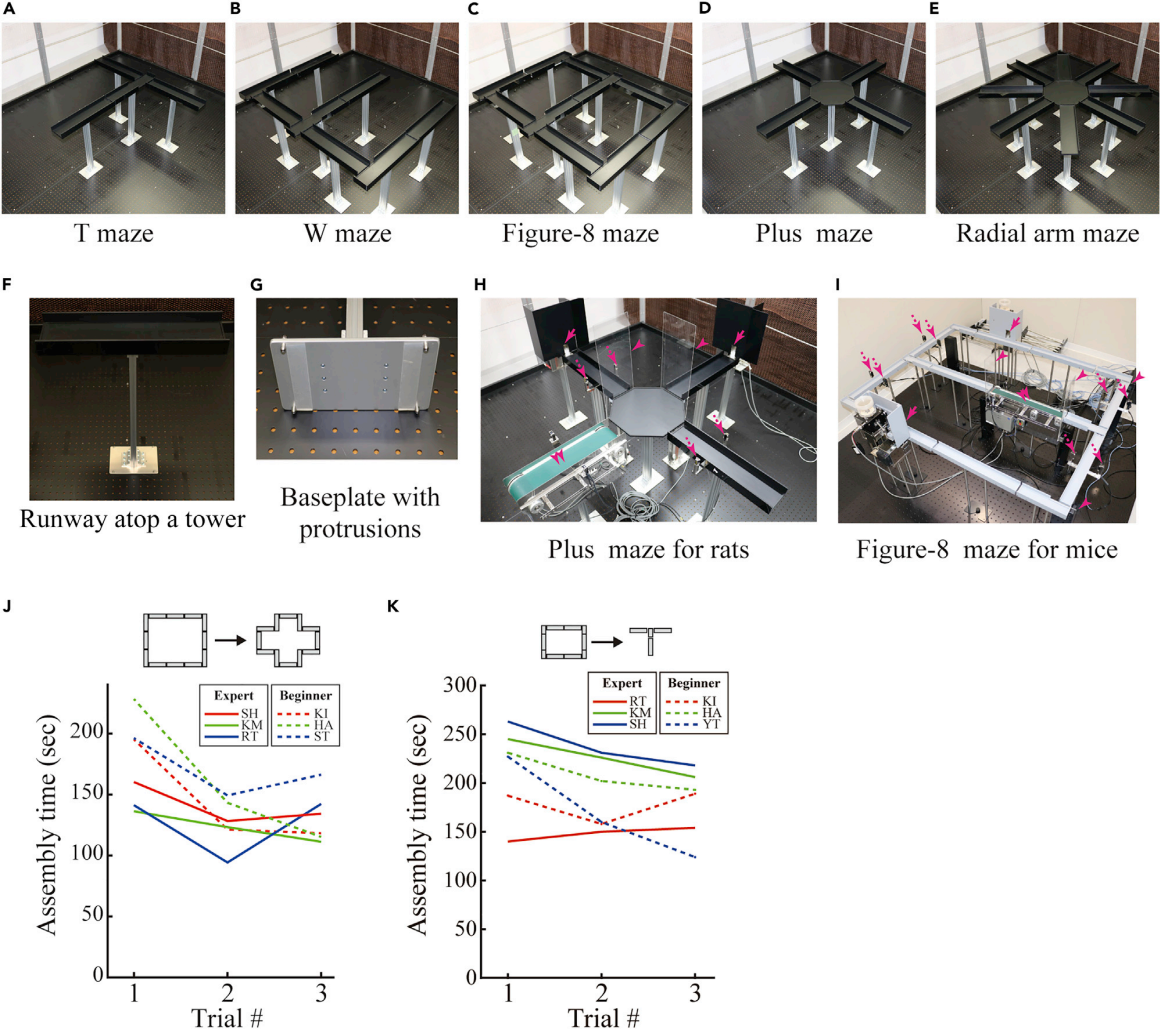
The Reconfigurable Maze For a Figure360 author presentation of this figure, see https://doi.org/10.1016/j.isci.2019.100787. (A–E) The maze can be configured to form T (A), W (B), figure-8 (C), plus (D), and radial arm (E) mazes in a single enclosure. (F) Runway sections are placed atop towers. (G) The baseplate of each tower has four protrusions that coordinate the placement of the section on the breadboard in a flexible, repeatable way. (H) Configured plus maze for rats with two feeders (arrows), two movable walls (arrowheads), one treadmill (double arrowhead), and two shut-off sensors (dashed arrows). (I) Small version of the reconfigurable maze for mice, configured as a figure-8 maze with two feeders (arrows), two movable walls (arrowheads), one treadmill (double arrowhead), and two shut-off sensors (dashed arrows). (J) Assembly time of the rat version of the reconfigurable maze for morphing the shape from square to cruciform. Performance improved with consecutive trials in a day (performance versus trial: *F*_2,8_ = 7.453, p = 0.0149), but not with expertise (expert/beginner) (performance versus expertise: *F*_1,4_ = 5.654, p = 0.0762). There was no significant interaction between expertise and experience in a day (expertise versus trial: *F*_2,8_ = 0.320, p = 0.735). Two-way mixed ANOVA was used. (K) Assembly time of the mouse version of the reconfigurable maze for morphing the shape from a rectangle to a T. There was no significant difference between performance and consecutive trials in a day (performance versus trial: *F*_2,8_ = 3.997, p = 0.0625) or expertise (expert/beginner) (performance versus expertise: *F*_1,4_ = 0.351, p = 0.58). There was no significant interaction between expertise and experience in a day (expertise versus trial: *F*_2,8_ = 0.658, p = 0.543). Two-way mixed ANOVA was used.

We developed a maze system that enables the reconfiguration of the shape of the maze in a single physical environment using interlocking runways. Figure 1 demonstrates the existing standard mazes configured by our maze system in an enclosure, namely, the T-maze, W-maze, figure-8 maze, plus maze, and radial arm maze. The runways are each placed atop towers with baseplates (Table S1); these baseplates have protrusions that connect to a grid of holes in a floor-based breadboard (Figure 1F). The insertion of the protrusions into the breadboard connection enables coordination with the grid pattern and minimizes the swinging of the runways resulting from the movement of the animal (Figure 1G). Similarly, an array of accompanying parts, including feeders, movable walls, and treadmills placed atop towers of their own, can also be attached on the breadboards. Thus, the feeder can be placed by the side of any runway to change the reward location (Figure 1H, arrows). The movable wall can be placed at any of the interlocking gaps between runways as a dead end to dynamically control possible running paths (Figure 1H, arrowheads), and any runway can be replaced by the treadmill (Figure 1H, double arrowheads). Moreover, the shut-off sensor can be attached alongside any runway to signal a triggering event to the feeders and treadmills (Figure 1H, dashed arrows). Placing these interlocking parts onto the grid pattern enables the experimenters, even if they are not familiar with the maze, to precisely reproduce a variety of coordinated patterns of mazes for a brief period in a repeatable manner in a single physical environment. The maze also provides a scalable experimental setup because the complexity and the area of the maze is incrementally expandable by adding extra parts. When the morphing of a maze from square to cruciform was timed the time it took beginners to assemble a maze was not significantly different from the assembly time of experts who had used the reconfigurable maze daily over 3 months (3 experts: 131 ± 9 s; 3 beginners: 158 ± 12 s [mean ± SEM]; Figure 1J, Video S1, S2, S3, S4, S5, and S6). Runway sections with tall sidewalls (35 cm height) for reducing fear can be placed to test anxiety (Figure S1). Instead of runways, open platforms can also be embedded into the maze, which enables the plus maze and radial arm maze to be configured (Figures 1D and 1E). Furthermore, the scheduler for the timing of sensors and actuators installed in the accompanying parts allows the experimenter to design several behavioral tasks, even within a single maze shape. Such flexible maze design also allows experimenters to rapidly select the tasks best matched to the needs of a particular experiment during preliminary studies. In accordance with this flexible design principle, we also developed a small version of the reconfigurable maze system for mice (Figure 1I, Table S2). Using the mouse version, we could also configure existing standard mazes, namely, the T-maze, W-maze, figure-8 maze, and plus maze (Figure S2). The assembly time for morphing a maze shape from square to T between experts and beginners was not significantly different (3 experts: 204 ± 15 s; 3 beginners: 186 ± 11 s [mean ± SEM]; Figure 1K), further demonstrating the usability of our system for screening behavioral phenotypes in mice.

Video S1. Morphing of a Maze from Square to Cruciform by S.H. (Expert), Related to Figure 1

Video S2. Morphing of a Maze from Square to Cruciform by K.M. (Expert), Related to Figure 1

Video S3. Morphing of a Maze from Square to Cruciform by R.T. (Expert), Related to Figure 1

Video S4. Morphing of a Maze from Square to Cruciform by K.I. (Beginner), Related to Figure 1

Video S5. Morphing of a Maze from Square to Cruciform by H.A. (Beginner), Related to Figure 1

Video S6. Morphing of a Maze from Square to Cruciform by S.T. (Beginner), Related to Figure 1

### Interlocking Gaps Do Not Alter Navigational Behavior

**Figure 2:**
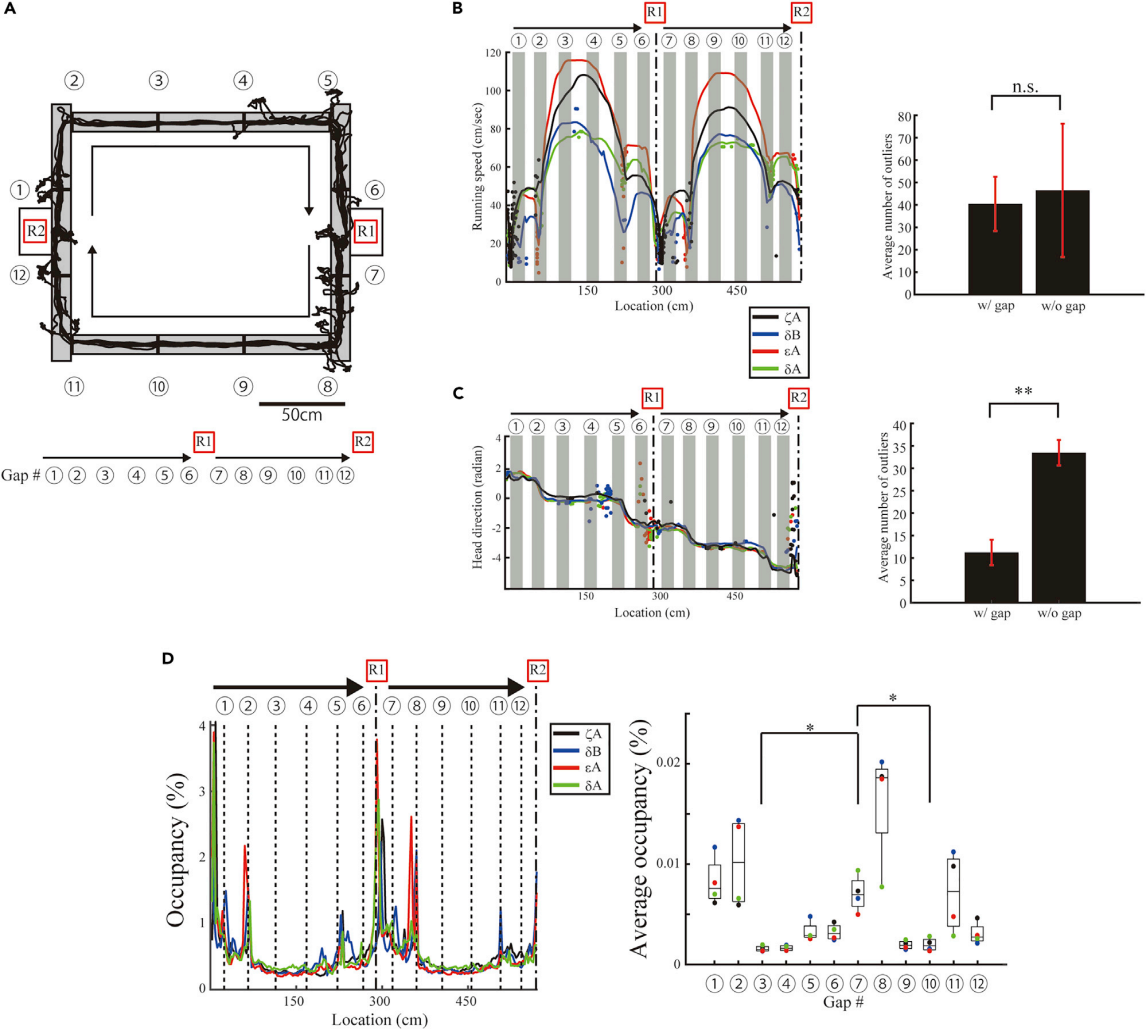
Interlocking Gaps Do Not Alter Navigational Behavior (A) Schematics of the square-shaped maze test. An example running trajectory of a rat is superimposed on the maze. The numbers indicate gap locations. R1 and R2 indicate food dispensers. Linearized gap locations are illustrated at the bottom. (B and C) Left, the average running speed and head direction of four rats as a function of the linearized location. The dots indicate the location of outliers for each lap. Right, the average number of outliers on the regions around the gap (gray shaded areas) and others (running speed: t = −0.19, df = 6, p = 0.86; head direction: t = −5.53, df = 6, p = 0.0015, two-tailed paired t test). **p < 0.01, n.s.: p > 0.05. Error bars indicate SEM. (D) Left, the percentage of the occupancy time over the entire maze as a function of the linearized location of four rats. Right, the median percentage of the occupancy time at gap locations (median, first and third quartiles, minimum, and maximum indicated). The rats preferentially slow down at gap #7 as compared with gaps #3 and #10, which are located at the top and bottom of the maze (F_1,3_ = 152.69, p = 0.0011; gap #7 versus #3: p = 0.049; gap #7 versus #10: p = 0.030, One-way repeated-measures ANOVA with post hoc Tukey's honestly significant difference test). *p < 0.05

To manufacture the interlocking parts at a reasonable cost or by hand, a short gap between runways (∼1 cm) (Figure 2A) is a prerequisite margin for the parts to interlock. Ideally, perfectly manufactured parts with a precision under 1 mm would not require these gaps as margins; however, such high-precision manufacturing is expensive and does not permit the use of low-precision handcrafted parts. When the gaps are not included as a margin, runways manufactured with lower precision (>1 cm) will overlap if the location of the runway is only slightly shifted, as shown in Figure S3.

We next answered the question of whether this gap affects animal behavior. Four rats were trained to smoothly run along a square-shaped maze in a clockwise direction. The running speed at the gaps did not abruptly change from the running speed between gaps (Figure 2B). Incidences of abrupt changes in the head direction were significantly lower at the gaps than between gaps (Figure 2C). These results suggest that the gaps did not distort normal rats' behaviors. To corroborate the result, we prepared two gapless runways the total lengths (149 cm long) of which were the same as those of the three interlocking runways, including the gaps between the sections. We then replaced three runway sections at the top or bottom of the square-shaped maze with a single runway without gaps (Figure S4A). Five rats were trained to run on the mazes, with or without gaps, over a 20-min interval. Both running speed and head direction on the gaps were not significantly different from those on the corresponding portions of the single runway without gaps (Figures S4B and S4C).

As shown from the trajectory (Figure 2A) and abrupt changes in the head direction (Figure 2C), the rats appeared to frequently exhibit exploratory behaviors at preferred locations (Hu and Amsel, 1995). To examine whether such exploration is preferentially observed at gap locations, four rats were trained to run in a clockwise direction on a square-shaped maze configuration. All rats sometimes paused and exhibited exploratory behaviors around food dispensers or corners. Indeed, the occupancy time when the rats visited the gap location around a food dispenser (gap #7) was significantly longer than the occupancy time on two other gaps (gaps #3 and #10) (Figure 2D), suggesting that unidentified factors, excluding the gap presence, might also affect rats' behaviors around food dispensers or corners. In addition, it is well known that such exploratory behaviors modulate hippocampal neuronal activity (Gauthier and Tank, 2018). Thus, to exclusively examine the gap effects, we focused on the gaps (#3, #4, #9, and #10) between the three interlocking runways arranged in a straight line on the top and bottom sides of the square-shaped maze in the subsequent analyses.

### Interlocking Gaps Do Not Alter Hippocampal Place Coding

**Figure 3:**
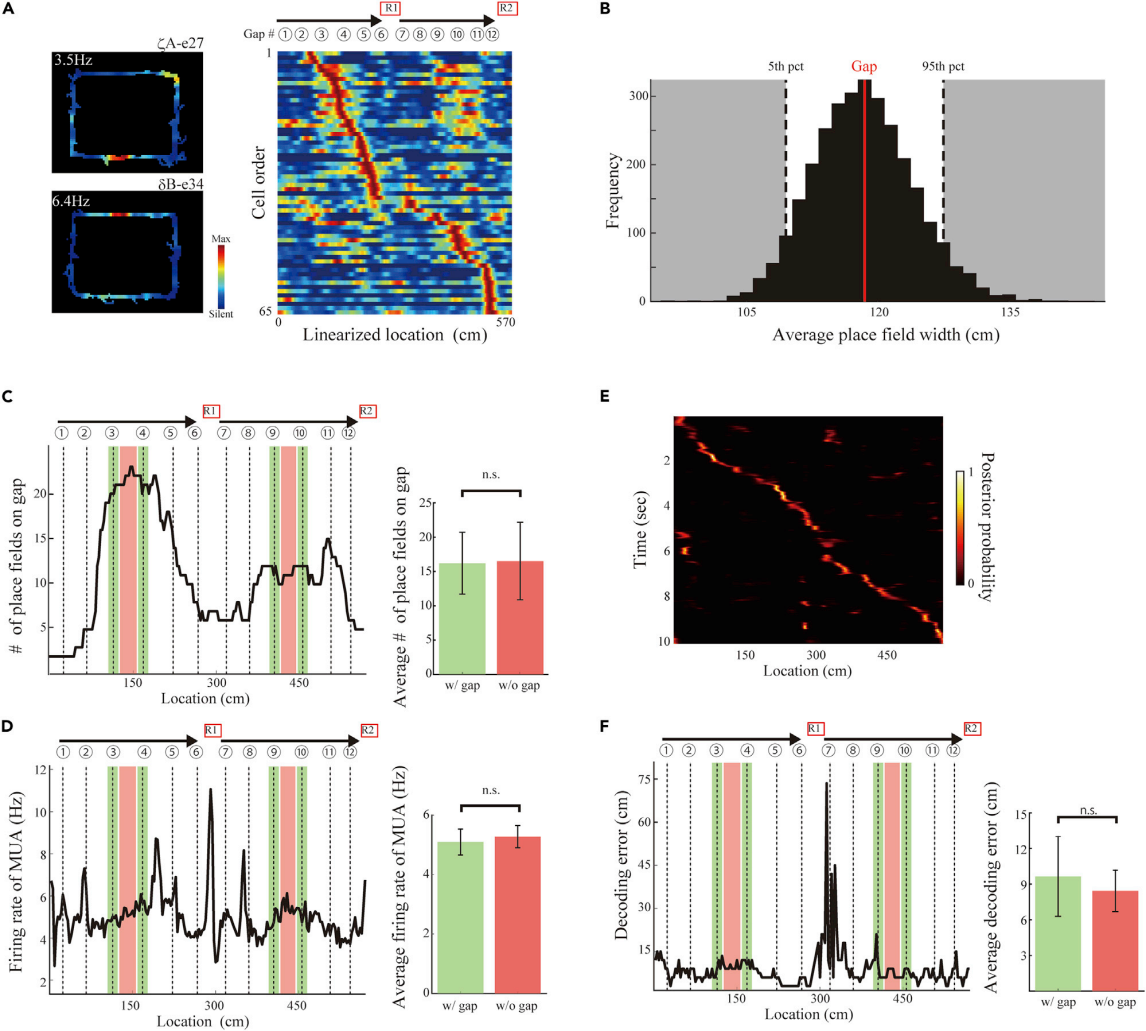
Interlocking Gaps Do Not Alter Hippocampal Place Coding (A) Left, normalized firing rate map of two representative place cells that have place fields around gap locations in the hippocampal CA1 on the square-shaped maze configuration. Right, normalized firing rate maps of 65 hippocampal place cells simultaneously monitored from a rat (ζA) ordered by the latency of their peak firing rates. Each line is a single unit. Gap locations are indicated at the top. Red indicates maximum firing rates, and blue indicates silent. (B) Distribution of the average width of place fields randomly shuffled from the original sample covering the gaps (3,000 shuffles). Red line indicates the average width of the place fields on the gaps. Dotted lines indicate 5^th^ and 95^th^ percentiles for the shuffled data. (C and D) Left, the number of place fields (C) and the firing rate of multi-unit activity (MUA) in the hippocampal CA1 (D) as a function of linearized location on the square-shaped maze. Right, the average number of place fields (C) (z = 0.14, p = 0.89) and the average firing rate of MUA (z = −1.51, p = 0.13) on the gaps (#3, 4, 9, 10) (green shaded area) and between them (orange shaded area). (E) Representative posterior probability of locations decoded by the Bayesian decoder from 89 simultaneously monitored cells. Values are indicated by the color bar (right). (F) Left, the difference between actual and decoded locations as a function of linearized location on the square-shaped maze. Right, the average difference between actual and decoded locations on the gaps (#3, 4, 9, 10) (green shaded area) and between them (orange shaded area) (z = 1.34, p = 0.18). Bin width is set at 3 cm. All error bars indicate SEM. All were from two-tailed Wilcoxon rank-sum test, n.s.: p > 0.05.

To determine whether the gaps distort neuronal activity in terms of spatial navigation, we monitored the activity of 236 neurons from the hippocampal CA1 of both hemispheres of four rats running on the square-shaped maze in a clockwise direction (Figure 3A, Table S3). The average width of place fields of 62 place cells on the gaps settled between the 5^th^ and 95^th^ percentiles of distribution for shuffled data from the entire set of place fields (Figure 3B). Moreover, the number of place fields from the 62 place cells and the firing rate of multiunit activity in the hippocampal CA1 on the gaps did not change when compared with recordings taken between the gaps (Figures 3C and 3D). At the ensemble level, the trajectory decoded from the activity of simultaneously monitored place cells using a memoryless Bayesian decoder (Zhang et al., 1998) depicted a seamless path even on the gap locations (Figures 3E and 3F). Thus, both behavioral and neuronal responses at the gaps support the view that rats behave as if they perceive the interlocking gap and gapless portions on the runways similarly.

### Rats and Mice Can Learn Spatial Alternation Tasks in the Reconfigured Maze

**Figure 4:**
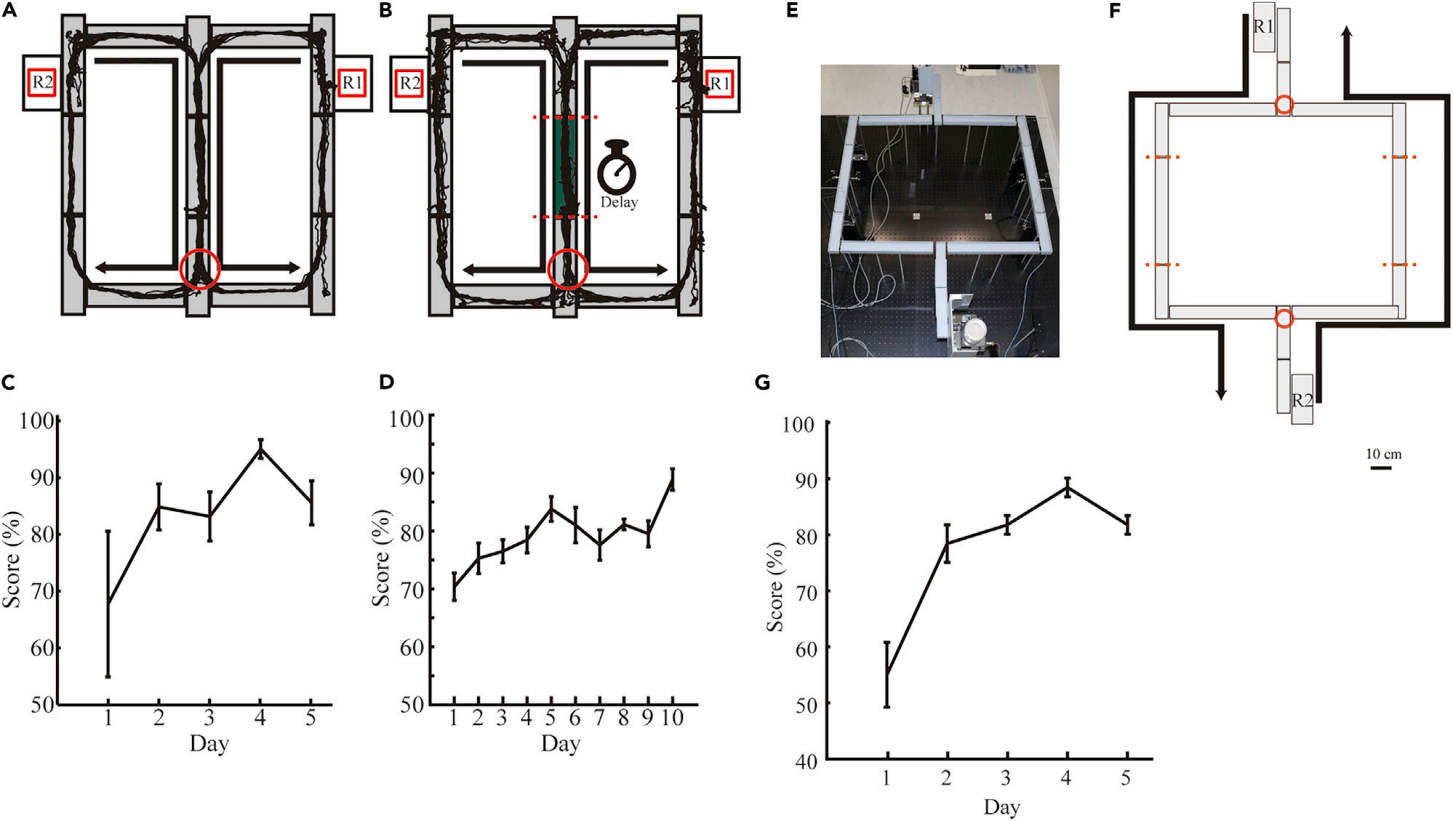
Rats and Mice Can Learn to Navigate the Configured Mazes (A) Schematic of the figure-8-shaped maze created using the rat version of the reconfigurable maze. Example animal trajectories are superimposed on top of the maze. The red circle indicates a branchpoint where the rat must decide which direction to turn. (B) Same as in (A) but with a treadmill (green). Red dashed lines indicate the gap locations where movable walls are placed to force the rats to run on the treadmill for a delay period. (C) Spatial alternation task performance improved with experience (performance versus testing day: F_1, 4_ = 795.9, p < 10^−5^). (D) Performance of the delayed version of the spatial alternation task of rats improved with experience (performance versus testing day: F_1,2_ = 531.8, p = 0.0019). All were from one-way repeated-measures ANOVA. Error bars indicate SEM. (E) Top view of the configured double T-maze using the mouse version of the reconfigurable maze. (F) Schematics of the double T-maze. Example mouse trajectories are superimposed on the maze. Arrow marks the running directions from the food dispensers (R1/R2). The red circles indicate branch points where the mice must decide which direction to turn. Red dotted lines indicate the locations where the movable walls are placed to prevent a reverse run. (G) Spatial alternation task performance of mice improved with experience (performance versus testing day: F_1,2_ = 7320, p = 0.000136). One-way repeated-measures ANOVA was used. Error bars indicate SEM.

After the five rats became familiar with the square-shaped maze, the maze shape was morphed from square to figure-8 using the reconfigurable maze system. The rats were trained to alternate between left and right at the branchpoint of the central stem for 1 h per day over 5 days (Figure 4A). All rats gradually learned the spatial alternation task (Figure 4C). They achieved a mean score of greater than 80% correct choices over 50 consecutive trials on the second day. To demonstrate the usability of the attachable walls and treadmills, three rats were then tested on a delayed version of the spatial alternation task by partially reconfiguring the maze: a runway in the center of the stem in the figure-8-shaped maze was replaced by a treadmill (Figure 4B). To force the rats to run on the treadmill for a 7-s delay period, movable walls were set at the gaps in front and behind the treadmill. The rats achieved a mean score of greater than 70% correct choices over 50 consecutive trials at the beginning, and their performance improved after 10 days of testing (Figure 4D).

After three mice were familiar with the linear track and rectangular maze in the mouse version of the reconfigurable maze, the maze shape was morphed to a T-maze (Figures 4E and 4F). The mice were trained to alternate between left and right at two branch points for 1 h per day over 5 days. All mice gradually learned the spatial alternation task (Figure 4G). They achieved a mean score of greater than 75% correct choices over 20 consecutive trials on the second day. These results and maze reconfigurations demonstrate that the reconfigurable maze system can consistently replicate the existing standard mazes and serve as tests of spatial learning and memory.

### Place Field Remapping during the Maze Morphing Experiment

**Figure 5:**
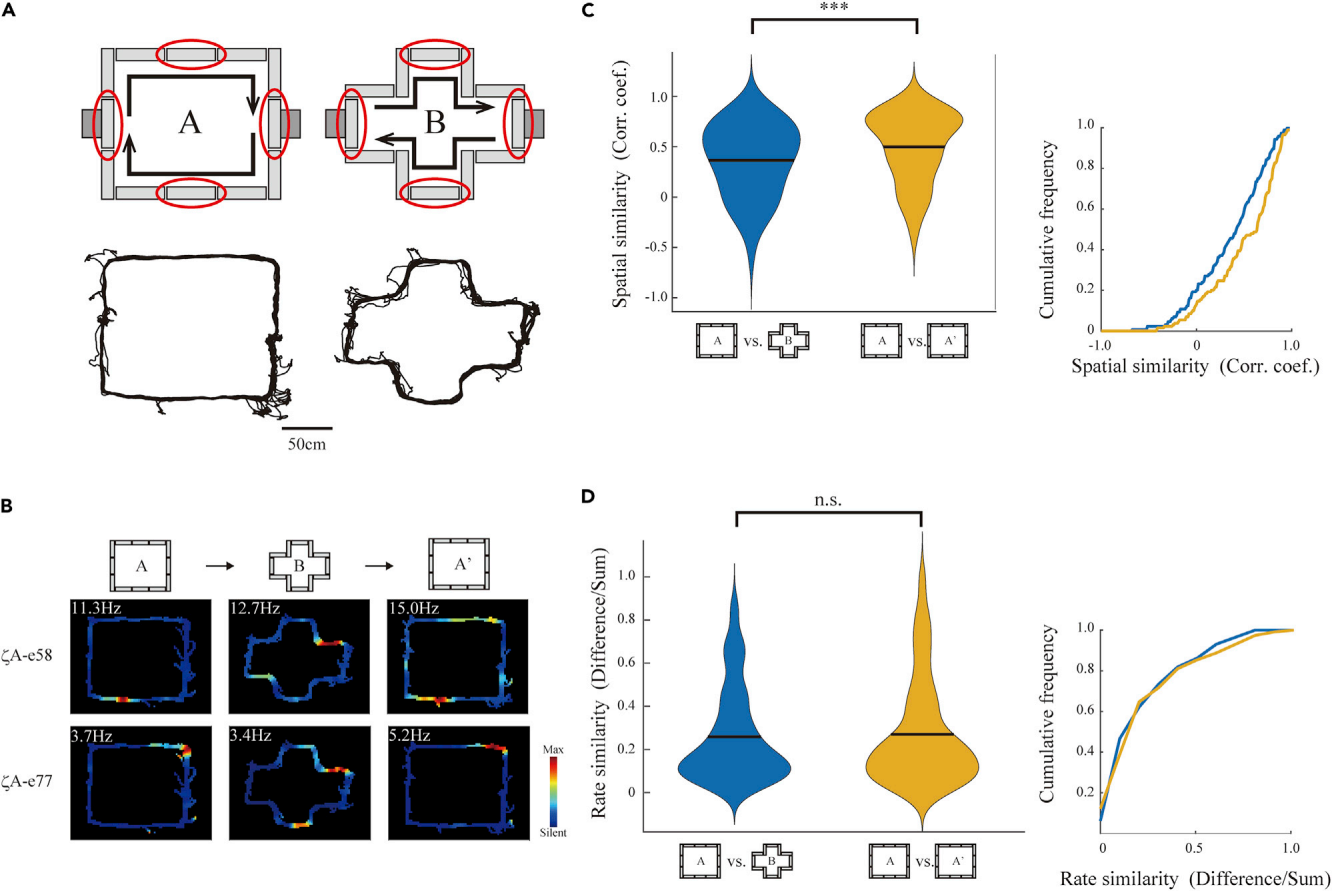
Place Field Remapping during the Maze Morphing Experiment (A) Schematics of square and cruciform mazes configured by our system (top) and the representative running trajectory of a rat (bottom). The runways enclosed by red circles were not moved during the morphing experiment. (B) Representative change of the place field of a place cell recorded from a rat (ζA) during maze morphing. The maximum firing rate is displayed at the top left corner. (C and D) Violin plots of spatial similarity as the spatial correlation of the firing rate map (C) (z = −3.98, p = 10^−4^) and rate similarity as firing rate difference within place fields (D) (z = −0.60, p = 0.55) between the square maze and cruciform maze, and between the first and second exposures of the square maze (left). The bar indicates the median. Surrounding each side is a rotated kernel density plot. Their cumulative frequency is graphed to the right. All were from two-tailed Wilcoxon signed-rank test. ***p < 0.001, n.s.: p > 0.05.

Several lines of evidence suggest the hippocampal place coding partially or completely changes in response to external or internal cues (Muller and Kubie, 1987, Leutgeb et al., 2005). This phenomenon is called the remapping of place fields. To demonstrate the repeatability and reproducibility of our reconfigurable maze system over existing mazes, we provide here a novel morphing experiment to examine the remapping of place fields between different shapes of a maze with an identical path length when the maze shape morphs from square to cruciform to square. As the cruciform maze is an internal structure of the square maze, the total path length does not change during morphing. Moreover, the four outermost runways of the cruciform maze physically overlap with the path of the square-shaped maze. As our maze realizes the morphing in one enclosure, it enables the identification of factors influencing hippocampal place coding: path integration and spatial reference frame (Figure 5A).

To examine the remapping of the place fields, we examined 129 place cells monitored from the dorsal hippocampal CA1 of four rats. The total path length of the runway and available external landmarks in the mazes were nearly identical across maze shape morphs, whereas the spatial correlation of the firing rate map of place cells within the overlapping runways in the square-shaped maze between the first and second exposures was significantly larger than that between the square-shaped maze and the cruciform maze (Figures 5B and 5C). This suggests that the maze shape can be a cue capable of inducing remapping of the location of place fields as a spatial reference frame. Moreover, the difference in the maximum firing rate of place fields within the overlapping runways in the square-shaped maze between the first and second exposures was similar to that between the square-shaped maze and the cruciform maze (Figures 5B and 5D). These results suggest that maze morphing from square to cruciform is represented by a difference in place field locations without a change in place field firing rate.

## Discussion

We demonstrate our reconfigurable maze system's ability to provide flexible, scalable, repeatable, and reproducible tests by configuring standard existing mazes in a single real environment and by examining spatial navigational behavior and neuronal activity during learning and memory performances in the mazes. Our maze system has the potential to become an invaluable, scalable tool for the study of learning and memory, including working and reference memory, spatial navigation, and decision-making, as well as anxiety. Using the reconfigurable maze, experimenters can consistently replicate a variety of mazes in their laboratory, and the position of the maze parts can be reconfigured for a brief period in a repeatable manner to test several task performances in a single physical environment.

Currently, a variety of maze tests are routinely used to gain a greater understanding of learning and memory in animals and for screening behavioral phenotypes in animal models of diseases. Although evidence is being accumulated, it is not necessarily available to contribute to our understanding of learning and memory mechanisms in the brain, because the underlying neuronal signatures are not necessarily similar, even when identical maze shapes are used across different conditions. For instance, if a behavioral phenotype for spatial memory deficits emerged in two maze tests, each conducted in a different room, the tests may only specifically show the remapping of hippocampal place coding across the different rooms, as demonstrated in the maze morphing experiment of the present study. Indeed, recent studies suggest that place cell activity sequences are linked to future planning (Pfeiffer and Foster, 2013) and episodic-like memory retrieval (Takahashi, 2015), as well as neurodegenerative diseases (Cheng and Ji, 2013). Therefore, such neuronal dissociations should be minimized in a comparative study across maze tests. Place cells show an irregular firing pattern in passive VR experiments (Aronov and Tank, 2014). Therefore, we designed our maze based on the concept that various shapes can be constructed in a single real environment, where several types of learning and memory tests can be performed. Recently developed active VR can markedly minimize such dissociations, specifically for proximal cues (Aronov and Tank, 2014, Stowers et al., 2017). Although our maze system and the active VR are not exclusive, the combination will enable the production of unlimited experimental situations without the neuronal distortions arising from both proximal and distal cues in the near future.

Our reconfigurable maze system is compatible with techniques for monitoring neuronal activity such as extracellular multiple single-unit recording (Wilson and McNaughton, 1993) and single-photon calcium imaging (Ziv et al., 2013) for freely behaving rats or mice. As demonstrated by the remapping of place fields across morphed mazes, the combination must be accelerated to allow understanding of the neuronal underpinning of learning and memory. Furthermore, our maze enables rapid prototyping of tasks by reconfiguring the coordination of the parts, including runways, rewards, and obstacles, within a few minutes, accelerating studies on learning and memory.

The reconfigurable maze is thus a promising maze platform for testing the performance of learning and memory, including working, reference and episodic memory, decision-making, spatial navigation, and anxiety, as well as for screening behavioral phenotypes of mice, including transgenic models of disease.

### Limitations of the Study

The initial setup cost and laboratory space for introducing the reconfigurable maze may be barriers to widespread distribution. However, the scalable features minimize these barriers. For instance, a minimal setup realized by removing optional modules from the full configuration (demonstrated by a square-shaped maze without movable walls or shut-off sensors) will reduce the cost. In addition, the modular architecture allows the configuration of small mazes to fit limited laboratory space. Although the minimal setup may not be a compelling reason for laboratories to invest in the reconfigurable maze, the complexity can be expanded incrementally by adding supplementary parts. Moreover, the setup and validation for novel configured mazes within a laboratory may be time consuming. Provided that the reconfigurable maze will be widely distributed and standardized, the overall cost will be lowered and information resources describing how to set up new configurations of the maze and validate their performance for specific aims will be shared between researchers. In this way, implementation of the reconfigurable maze will ensure reproducibility and repeatability.

Despite its flexibility, reproducibility, repeatability, and scalability, our maze has a few limitations. Although oblique runways at an angle of 45° could be embedded into our maze as demonstrated by the configured radial arm maze (Figure 1E), the angle cannot be flexibly modified because the coordinated grid assignment of the parts requires the runways to be placed at a fixed angle. Unlike the recently developed honeycomb maze (Wood et al., 2018), the tower supporting the runway cannot be raised and lowered automatically. Although adjustable mechanisms are capable of overcoming those limitations, they increase the damage rate of the physical system during behavioral and neuronal recordings, reducing the major advantage of reproducibility and repeatability.

## Methods

All methods can be found in the accompanying Transparent Methods supplemental file.

## Data and Code Availability

The detail of the maze parts is freely available (https://github.com/TakahashiLab/ReconfigurableMazeParts). The controlling software is also freely available (https://github.com/TakahashiLab/ReconfigurableMazeGUI).

## Supplemental Information

Document S1. Transparent Methods, Figures S1–S4, and Tables S1–S3

Figure360. An Author Presentation of Figure 1
