# Supplementary material for: The Reconfigurable Maze Provides Flexible, Scalable, Reproducible, and Repeatable Tests
Source: iScience. 2019 Dec 19;23(1):100787. doi: 10.1016/j.isci.2019.100787 (PMC6992939; doi:10.1016/j.isci.2019.100787)
Supplement: Document S1. Transparent Methods, Figures S1–S4, and Tables S1–S3 [file mmc1.pdf]

iScience, Volume *23*

Supplemental Information

The Reconfigurable Maze Provides Flexible, Scalable, Reproducible, and Repeatable Tests

Satoshi Hoshino, Riku Takahashi, Kana Mieno, Yuta Tamatsu, Hirotsugu Azechi, Kaoru Ide, and Susumu Takahashi

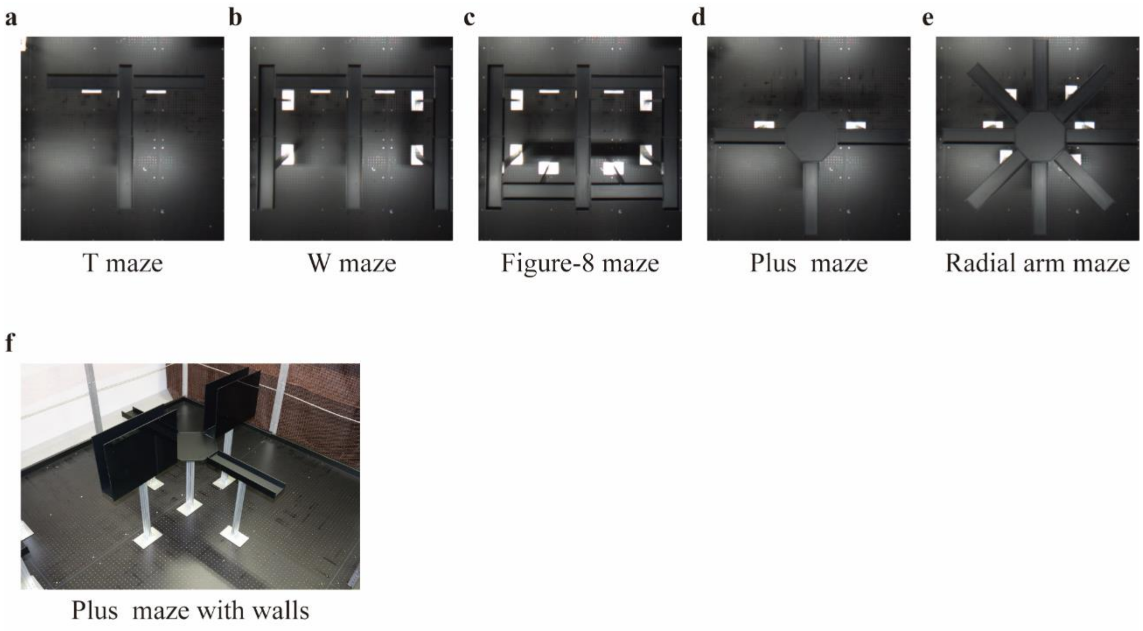

Figure S1. Top view of configured mazes, Related to Figure 1. (**a**–**e**) Top view of maze shape configured to T (**a**), W (**b**), figure-8 (**c**), plus (**d**), and radial arm (**e**) mazes in an enclosure. This view demonstrates that several shapes of the maze can be configured in the same space. (**f**) The plus maze configured with tall sidewalls for testing anxiety.

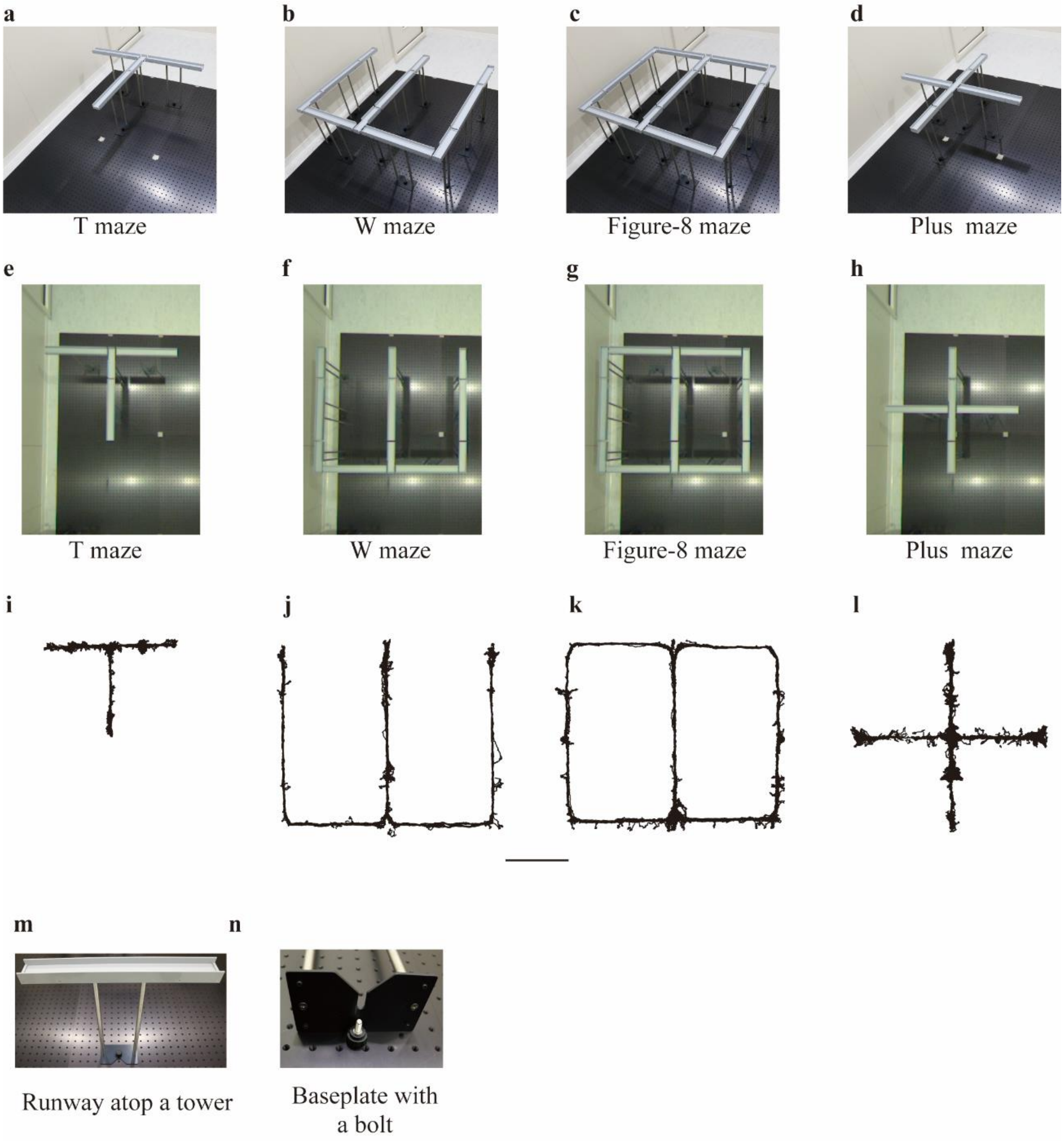

Figure S2. The reconfigurable maze for mice, Related to Figure 1. (**a**–**d**) The shape was configured to match T (**a**), W (**b**), figure-8 (**c**), and plus (**d**) mazes in an enclosure. (**e–h**) Top view of the mazes. Note that the central stem remains in the same position. (**i–l**) Example running trajectory of a mouse in each maze. Scale bar, 40 cm. (**m**) The runway is placed atop a tower on a breadboard with a grid of holes. (**n**) The baseplate of the tower is fixed by a bolt because the weight of the baseplate alone is too light to support the runway. The grid of the breadboard allows the flexible coordination of the runways and accessory parts in repeatable ways.

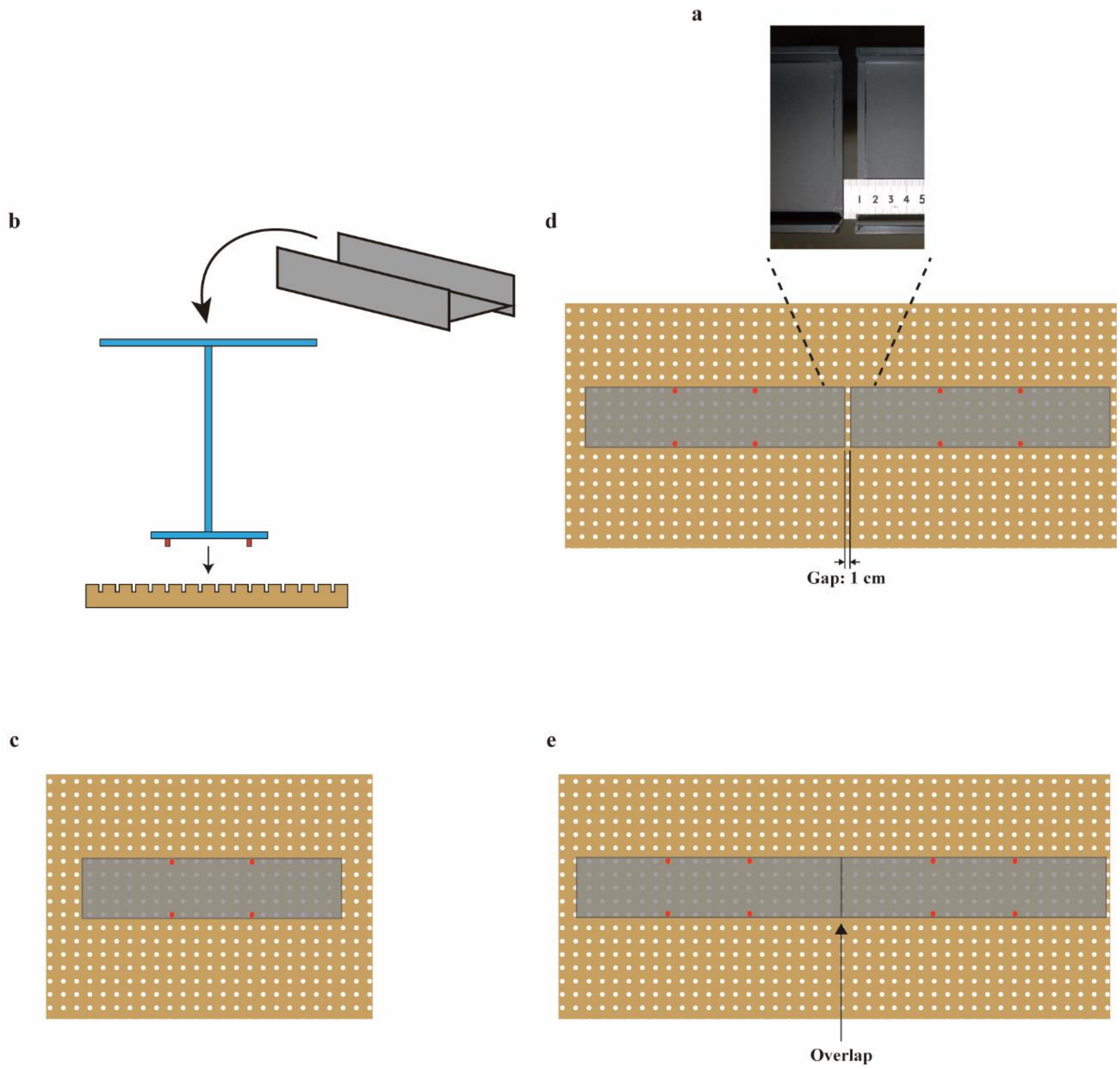

Figure S3. A short gap between runways is a prerequisite margin for the interlocking parts, Related to Figure 1. (**a**) Close-up photo showing the gap. (**b**) The runway (gray) is placed atop a tower (blue) on a breadboard (brown) with a grid of holes. The baseplate of the tower has protrusions (red) that coordinate the placement of the section on the breadboard. (**c**) Top view of a runway on the breadboard. (**d**) Two runways with a 1 cm gap. (**e**) This view demonstrates that the runways overlap only if the total dimensional error exceeds a precision of 1 cm.

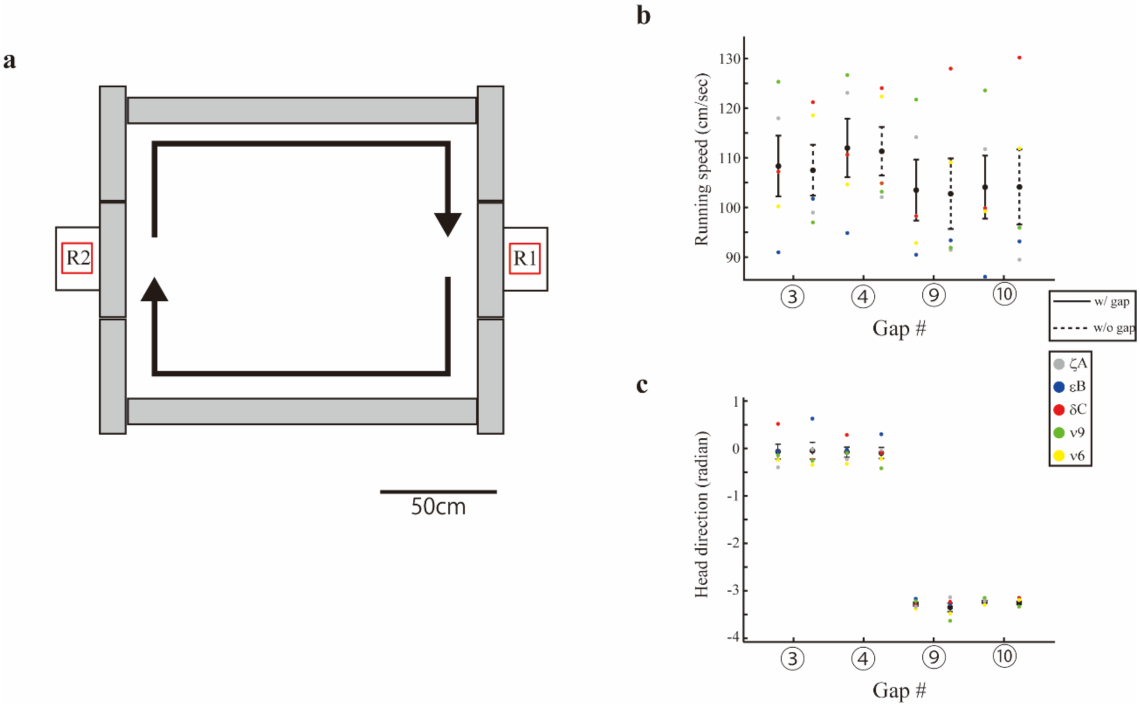

Figure S4. The running speed and head direction on the runway with or without gaps, Related to Figure 2. (**a**) Schematic showing two gapless runways placed at the top and bottom of the square-shaped maze. R1 and R2 indicate food dispensers. (**b–c**) The running speed (**b**) and head direction (**c**) of five rats on the runways with gaps (solid line) and without gaps (dotted line) as a function of the examined gap locations. The data from individual animals are overlapped as color-coded dots. Neither running speed nor head direction were influenced by the presence of a gap (simple main effect: gap presence vs. running speed between all pairs of the examined gaps: $P > 0.05$, gap presence vs. head direction between all pairs of the examined gaps: $P > 0.05$). However, there were interactions between gap presence and location (running speed: $F_{1,4} = 2653.9$, $P < 10^{-6}$; head direction: $F_{1,4} = 302.3$, $P < 10^{-4}$) shown using two-way repeated-measures ANOVA. Error bars indicate SEM.

Table S1. Dimensions of the runway for the rat version of the reconfigurable maze, Related to Figure 1.

Runway type	Combination
Straight	A, B, C, D, E
Right	C, F, G, H, I, J, K, L, T
Left	C, F, G, H, I, J, K, L, T
Central	C, F, H, I, J, K, L, T
S-shaped	H, M, N, O, P
Right and Left	C, Q, R, S, T
Octagonal field	W, X, T
Long straight	C, U, V, T

Basic component	Polygon type	Depth (mm)	Width (mm)	Height (mm)
A	Rectangle	5	490	100
B	Rectangle	5	490	45
C	Rectangle	2	100	5
D	Rectangle	5	100	5
E	Rectangle	5	440	10
F	Non-regular	5	485	100
G	Rectangle	5	485	45
H	Rectangle	5	100	45
I	Rectangle	5	260	45
J	Rectangle	10	480	15
K	Non-regular	10	480	15
L	Non-regular	2	230	10
M	Non-regular	5	480	100
N	Rectangle	5	305	45
O	Non-regular	10	480	15
P	Non-regular	2	180	10
Q	Non-regular	5	490	100
R	Non-regular	2	330	10
S	Non-regular	10	450	15
T	Rectangle	5	100	10
U	Rectangle	5	685	100
V	Rectangle	5	100	10

		Depth (mm)	Side (mm)	
W	Octagon	5	140	
X	Non-regular	2	140	

Runways can be assembled using a combination of basic components (A-X). All 3D models are freely available (https://github.com/TakahashiLab/ReconfigurableMazeParts).

Table S2. Dimensions of the runway for the mouse version of the reconfigurable maze, Related to Figure 1.

Runway type	Combination
Straight	A, B, C, D
Right	A, B, C, D, G, H, I
Left	A, B, C, D, G, H, I
Right and Left	A, C, D, G, H, I
Central	A, C, D, E, F

Basic component	Polygon type	Depth (mm)	Width (mm)	Height (mm)
A	Rectangle	3	391	40
B	Rectangle	3	391	28
C	Rectangle	10	40	10
D	Rectangle	3	40	5
E	Rectangle	3	140	28
F	Rectangle	3	115	5
G	Rectangle	3	40	15
H	Rectangle	3	80	5
I	Rectangle	3	311	28

Runways can be assembled using a combination of basic components (A-I). All 3D models are freely available (https://github.com/TakahashiLab/ReconfigurableMazeParts).

Table S3. Variability between rats: electrophysiological measurements, Related to Figure 5.

RAT #	ζA	δB	δA	εA	Total
No. of pyramidal cells	89	40	46	35	210
No. of interneurons	8	4	9	5	26
Analyses for the square-shaped maze for gap effects					
No. of place cells (spatial information > 0.1 bit/spike & peak firing rate > 1 Hz)	28	18	14	2	62
Spatial information (bits/spike, mean ± sem)	0.41±0.05	0.30±0.03	0.66±0.13	0.22±0.06	
Unit isolation quality (isolation distance, Median ± QD)	8.3 ±5.5	5.9 ±12.2	14. 1 ±8.7	6.4 ±2.7	
Analyses for the maze morphing from square to cruciform to square					
No. of place cells (spatial information > 0.1 bit/spike & peak firing rate > 1 Hz in any of three situations)	60	24	28	17	129
Spatial information (bits/spike, mean ± sem)	0.67±0.11	0.24±0.03	1.06±0.17	0.96±0.34	
Unit isolation quality (isolation distance, Median ± QD)	8.3 ±5.6	6.7 ±6.2	6.9 ±8.0	6.8 ±3.6	

(*) The number of cells meeting the criteria had a large difference between the two conditions because some cells met the criteria either in the square-shaped maze or in the cruciform maze during morphing.

Supplemental Video legends

Supplemental Video 1. Morphing of a maze from square to cruciform by S.H. (expert), Related to Figure 1.

Supplemental Video 2. Morphing of a maze from square to cruciform by K.M. (expert) , Related to Figure 1.

Supplemental Video 3. Morphing of a maze from square to cruciform by R.T. (expert) , Related to Figure 1.

Supplemental Video 4. Morphing of a maze from square to cruciform by K.I. (beginner) , Related to Figure 1.

Supplemental Video 5. Morphing of a maze from square to cruciform by H.A. (beginner) , Related to Figure 1.

Supplemental Video 6. Morphing of a maze from square to cruciform by S.T. (beginner) , Related to Figure 1.

Transparent Methods

Maze system implementation. The reconfigurable maze consists of interlocking runways (49 cm × 10 cm for rats, see Table S1; 39 cm × 5 cm for mice, see Table S2) and an array of accompanying parts including feeders, movable walls, shut-off sensors, and treadmills. Each runway is placed atop a tower mounted on a breadboard with a grid of holes (hole-to-hole spacing: 25 mm for rats, 25 mm for mice), that enables each section to be mounted independently of other runway sections. For rats, each runway made of 5 mm thick black polyvinyl chloride (PVC) (matte finish) is 55 cm above the breadboard (Table S1). For mice, 3 mm thick gray PVC is 34 cm above the breadboard (Table S2). The tower and its baseplate are made of aluminum. For rats, the baseplate has four protrusions that can be inserted into the holes of the breadboard to attach it. For mice, a bolt was inserted into the holes because the weight of the baseplate was too light to support the runway. Sidewalls (45 mm height for rats; 30 mm height for mice) around the top of the runway prevent the rats and mice from slipping off the runway. The elevated runways prevent the rats and mice from jumping out of the maze. The maze sits within a shielded enclosure (4 m × 5 m for rats; 1.8 m × 3.0 m for mice) covered by a copper mesh. All metal parts are grounded to reduce electrical artifacts in the electrophysiological recording.

An Arduino Mega controller was used to receive signals from shut-off sensors and to send activation signals to the actuators in the treadmills and feeders according to the user-defined sensor and actuator schedule. Custom-made scheduling software written in Matlab was used to monitor the location of rat or mouse via shut-off sensors and to control the actuators in the treadmills and feeders, which enables the feeders to be turned on and off according to the location of rats or mice.

Animals. Ten Long–Evans rats and four C57BL/6J mice purchased from Shimizu Laboratory Supplies, Co. Ltd. (Kyoto, Japan) were housed individually in cages (20 × 25 × 23 cm for rats, 14 × 21 × 12 cm for mice) where the light was maintained on a 12-hour light/12-hour dark schedule with the light phase starting at 8:00 am. The tests were performed in the light phase. The weight of all rats or mice was kept at 80% of free-feeding body weight. To examine hippocampal place coding on the maze, a custom-made microdrive was implanted into the dorsal hippocampal CA1 of both hemispheres (eight tetrodes each) of four rats to record multiple single-unit activities. All procedures were approved by the Doshisha University Institutional Animal Care and Use Committees.

Surgery, electrode preparation, and recording. Under isoflurane anesthesia, a custom-made microdrive with 16 independently movable tetrodes was fixed to the skull above the

hippocampus of both hemispheres (eight tetrodes each; AP 3.8 mm, ML 3.0 mm, DV 0.5–1.0 mm) of four rats (Table S3). After surgery, the electrodes were individually lowered into the pyramidal cell layer of the dorsal hippocampal CA1. The extracellular signals were amplified, buffered, digitized, and continuously sampled at 25 kHz using two 32-channel RHD2000 chips (Intan Technologies, Inc., CA) via a motorized commutator (*AlphaComm-I*; AlphaOmega Inc., Israel). The spikes and local field potential (LFP) were digitally filtered at 800–7.5 kHz and 0.1–200 Hz, respectively. The occurrence of sharp-wave ripple events in the LFP during immobility periods was used to estimate the pyramidal cell layer. After spike sorting using *KlustaKwik*, putative principal cells were distinguished from fast-spiking cells based on average firing rate (5 Hz). We defined place cells if the following criteria were met: the overall firing rate was >0.1 Hz, spatial information was > 0.1 bit/spike, and maximum firing rate was >1.0 Hz.

Behavioral training. All rats and mice were food-restricted and reduced to 80% of their ad libitum body weight over a two-week period before training. During this time, they were handled daily. To train rats or mice to obtain pellets from the food dispenser, they were initially placed on a box (48 cm × 24 cm, 32 cm height for rats; 34 cm × 24 cm, 19.5 cm height for mice) with the food dispenser at a corner.

Rats. The rats were trained on L, C, or G-shaped mazes configured by the reconfigurable maze system in the testing enclosure where they were habituated to the sounds made by the movable walls being raised and lowered. The rats performed a small, square-shaped maze task (overall: 120 cm × 49 cm) in which they ran in a clockwise direction to obtain a pellet. The training lasted until the rat learned to obtain at least one pellet per minute within a 25-minute experimental period. Next, the rats trained to run in a clockwise direction in a large square-shaped maze (overall: 170 cm × 148 cm) to obtain pellets from two food dispensers located at the left and right sides of the maze. The training lasted for at least 25 minutes per day and continued until the criteria of at least one trial per minute was achieved over one week. After the initial training, rats were trained to perform in the morphing experiment and the spatial alternation tasks described below.

Mice. Unlike the rats, the mice were trained on a linear track with a movable wall where they obtained food pellets at both sides and were habituated to the sounds of the movable wall. The mice performed a rectangle-shaped maze task (overall: 49 cm × 80 cm). The training lasted until they learned to obtain at least one pellet per minute for 25-minute intervals. After the initial training, they were trained to run on the figure-8 shaped maze, double T-maze, plus maze, and W-maze to obtain food pellets.

Morphing experiment. Four rats were trained to run in a clockwise direction on the maze morphing from square to cruciform to square. They ran within ~1 hour in the following sequence:

square maze, cruciform maze, and square maze. The 15-minute long sequences were spaced at ~5-minute intervals, and the rats were rewarded each time they arrived at the food dispensers located at both the left and right sides of the maze. Each maze morphing was done within approximately 5 minutes. The unit recording was made after the rats had experienced the maze morphing over a few days.

Spatial alternation task.

Rats. Five rats were trained to alternate between left and right at a decision point on the figure-8 shaped maze until they achieved an 85% correct rating for 25 minutes. A delay period was then incorporated. During the delay period, rats were locked between two movable walls in front and behind a treadmill. The treadmill rotated at a constant speed (~20 m/min) during the delay period. The delay period was incremented from 1 sec to 7 sec every testing day.

Mice. Three mice were trained to alternate left and right at two decision points on the double T maze until they achieved a 75% correct rating for an hour (Figure 4g).

Animal trajectory and head direction. The tip and root of the head were tracked from images captured at 50 or 100 frames per second by a USB3.0 digital video camera mounted on the ceiling of the enclosure using *DeepLabCut* (Mathis *et al.*, 2018). Initially, 200 annotated images were used to train the pre-trained ResNet-50 network using transfer learning. A few additional iterations of training were then performed with the goal that all Euclidian distances between tracked locations in adjacent frames would be under 50 pixels. Running trajectory was reconstructed by concatenating the tracked root of the head. Head direction was computed from the tip and root of the head by the inverse of the tangent function.

Analyses. The rat's trajectory was linearized for each trial by projecting the actual trajectory onto a predefined idealized trajectory using nearest-neighbor Delaunay triangulation. Spatial bins had a resolution of approximately 3 cm.

Rate map. A firing rate map of well-isolated neurons was constructed in a standard manner by dividing the total number of spikes in a bin (3 cm × 3 cm) at a given location by the total amount of time that the rat has been in that bin. Each value was smoothed with a Gaussian filter with a variance of three.

MUA. MUA was calculated by summing the firing of all monitored cells including low-firing cells and fast-spiking cells.

Spatial information. The spatial information (bits per spike) was used to measure how much information a spike conveys about the rat's location on the maze (Skaggs *et al.*, 1993). This is calculated by the following formula:

$$Spatial\ information = \sum_{i} P_i \left(\frac{R_i}{R}\right) \log_2 \left(\frac{R_i}{R}\right)$$

where *i* indexes over the position bins, P_i is the probability that the rat was in bin *i*, R_i is the mean firing rate in bin *i*, and *R* is the overall mean firing rate. On the basis of the spatial information, we identified the place cells.

Spatial and rate similarity measures. The spatial similarity is expressed by calculating the spatial correlation of place fields between the spatially overlapping paths of the square and cruciform mazes. The difference in firing rates was expressed by calculating a difference/sum score (Leutgeb *et al.*, 2005). The unsigned difference between the two maximum firing frequencies was calculated, and the difference was divided by the sum of the two rates to obtain the score for a set of conditions during maze morphing. Both similarities were also expressed using the cumulative distribution function, f (X < x), which gives the probability that the variable X will be X < x for each real number x.

Bayesian decoding. A memoryless Bayesian decoder (Zhang *et al.*, 1998) was used to decode the rat's locations on the basis of place cell activity. First, the probability of an rat's location given place cell firings within a time window was estimated as follows:

$$\mathrm{Prob}(Pos \mid spikes) = \left(\prod_{i=1}^{N} f_i(Pos)^{n_i}\right) \ \exp^{-\tau \sum_{i=1}^{N} f_i(Pos)}$$

where f_i and n_i represent the place map and the number of spikes of the *i*-th place cell within the time window, respectively, N indicates the total number of place cells, and τ represents the duration of the time window.

The probability within each time window was normalized for every location as follows(Pfeiffer and Foster, 2013):

$$\mathrm{nProb}(Pos \mid spikes) = \mathrm{Prob}(Pos_k \mid spikes) \Big/ \sum_{k=1}^{M} \mathrm{Prob}(Pos_k \mid spikes)$$

where $\mathrm{Prob}(Pos_k \mid spikes)$ represents the probability at the *k*th location bin within the time window, and M represents the total number of location bins.

The time window was set at 300 ms. A point estimation of the location was made using the maximum likelihood estimation.

Statistical analyses.

Analyses of rat's behavior around the gaps. Any value of running speed or head direction that exceeded more than three times the local scaled median absolute deviations (MAD) away from the local median within a sliding window (~72 cm) was defined as an outlier. The two-tailed

Wilcoxon rank-sum test was used to assess the gap effect on rat's behaviors in terms of running speed and head direction (Fig. 2b–c). Differences in running speed and head direction between gap locations between runways and the corresponding locations on the long gapless runway were assessed by two-way repeated-measures ANOVA (Fig. S4b–c). Differences in occupancy time at gap locations were assessed by one-way repeated-measures ANOVA (Fig. 2d). The occupancy time was calculated as the duration when the rat occupied the gap location. The duration while the rat paused (running speed < 5 cm/s) was excluded from the analysis.

Analyses of neural activity around the gaps. To examine whether the width of the place field was specifically changed at the gap locations, the Monte Carlo method was used. For each cell, the original place field location was shifted by a pseudo-random interval between 20 bins and 20 bins less than the length of the entire linearized track, with the end of the track wrapped to the beginning. This procedure was repeated 3,000 times for each cell. For each shuffling, the width of place field that is defined as the length of the firing field whose firing rate is over one-third of its maximum firing rate was calculated. Whether the place field on the gaps had a width greater than the 5th percentile or lower than the 95th percentile of the shuffled data was examined (Fig. 3b). Two-tailed Wilcoxon rank-sum test was used to assess the number of place fields, firing rates of MUA, and Bayesian decoding errors on the gap locations as compared with those on the non-gap locations (Fig. 3c-d, f).

Analyses of learning performance. The differences in learning curves were assessed using one-way repeated-measures ANOVA (Fig. 4). Two-way mixed ANOVA was used to ascertain the effect of expertise and experience on assembly time, and their potential interaction (Fig. 1j-k). The difference in spatial and rate similarities was assessed using the two-tailed Wilcoxon signed-rank test (Fig. 5c–d).

Analysis software. All analyses were performed using custom-made programs based on Matlab functions (v9.6; MathWorks, Natick, MA).

Histology. To identify the final recording locations, four rats were deeply anesthetized with isoflurane (Pfizer Japan Inc., Tokyo, Japan) and then given an overdose of pentobarbital sodium salt (50mg/kg, intraperitoneal (i.p.); Nacalai Tesque Inc., Kyoto, Japan) and transcardially perfused with phosphate-buffered saline (PBS), followed by 10% phosphate buffered formalin fixative (3.5-3.8% formaldehyde). Their brains were cut coronally at 40 μm and stained with cresyl violet. The final location of the tip of each electrode was around or below the pyramidal cell layer of the dorsal hippocampal CA1.

Supplemental References

Leutgeb, S. *et al.* (2005) 'Independent codes for spatial and episodic memory in hippocampal neuronal ensembles.', *Science*, 309(July), pp. 619–623. doi: 10.1126/science.1114037.

Mathis, A. *et al.* (2018) 'DeepLabCut: markerless pose estimation of user-defined body parts with deep learning', *Nature Neuroscience*, 21(9), pp. 1281–1289. doi: 10.1038/s41593-018-0209-y.

Pfeiffer, B. E. and Foster, D. J. (2013) 'Hippocampal place-cell sequences depict future paths to remembered goals.', *Nature*, 497, pp. 74–9. doi: 10.1038/nature12112.

Skaggs, W. E. *et al.* (1993) 'An information-theoretic approach to deciphering the hippocampal code', *Advances in neural information processing systems*. Edited by S. J. Hanson, J. D. Cowan, and C. L. Giles, 5(2), pp. 1031–1037.

Zhang, K. *et al.* (1998) 'Interpreting neuronal population activity by reconstruction: unified framework with application to hippocampal place cells.', *Journal of neurophysiology*, 79, pp. 1017–1044.
